# Preparation and Characterization of Hydrogel Films and Nanoparticles Based on Low-Esterified Pectin for Anticancer Applications

**DOI:** 10.3390/polym15153280

**Published:** 2023-08-02

**Authors:** Aleksandra A. Patlay, Andrei S. Belousov, Vladimir E. Silant’ev, Roman A. Shatilov, Mikhail E. Shmelev, Valeri V. Kovalev, Irina V. Perminova, Ivan N. Baklanov, Vadim V. Kumeiko

**Affiliations:** 1 Institute of Life Sciences and Biomedicine, Far Eastern Federal University, Vladivostok 690922, Russia; patlay.al@mail.ru (A.A.P.); belousov.ands@gmail.com (A.S.B.); shatilov.ra@dvfu.ru (R.A.S.); shmelev.m.e@gmail.com (M.E.S.);; 2 Laboratory of Electrochemical Processes, Institute of Chemistry, Far Eastern Branch of Russian Academy of Sciences, Vladivostok 690022, Russia; 3 A.V. Zhirmunsky National Scientific Center of Marine Biology, Far Eastern Branch of Russian Academy of Sciences, Vladivostok 690041, Russia; 4 Department of Chemistry, Lomonosov Moscow State University, Leninskie Gory 1-3, Moscow 119991, Russia; iperm@med.chem.msu.ru

**Keywords:** nanocarrier, drug delivery systems, carbohydrates, polysaccharides, biomaterials, viscoelastic properties, blood–brain barrier, brain tumors, glioma, glioblastoma

## Abstract

Prospective adjuvant anticancer therapy development includes the establishing of drug delivery systems based on biocompatible and biodegradable carriers. We have designed films and nanoparticles (NPs) based on low-esterified pectin hydrogel using the ionic gelation method. We investigated morphology, nanomechanical properties, biocompatibility and anticancer activity. Hydrogel films are characterized by tunable viscoelastic properties and surface nanoarchitectonics through pectin concentration and esterification degree (DE), expressed in variable pore frequency and diameter. An in vitro study showed a significant reduction in metabolic activity and the proliferation of the U87MG human glioblastoma cell line, probably affected via the adhesion mechanism. Glioma cells formed neurosphere-like conglomerates with a small number of neurites when cultured on fully de-esterified pectin films and they did not produce neurites on the films prepared on 50% esterified pectin. Pectin NPs were examined in terms of size distribution and nanomechanical properties. The NPs’ shapes were proved spherical with a mean diameter varying in the range of 90–115 nm, and a negative zeta potential from −8.30 to −7.86 mV, which indicated their stability. The NPs did not demonstrate toxic effect on cells or metabolism inhibition, indicating good biocompatibility. Nanostructured biomaterials prepared on low-esterified pectins could be of interest for biomedical applications in adjuvant anticancer therapy and for designing drug delivery systems.

## 1. Introduction

One of the main problems of the therapy for oncological diseases is the targeted delivery of drugs directly to an affected tissue area that can reduce the negative effect on healthy cells during treatment [1]. Additional difficulties occur in the therapy for the brain diseases. Drug delivery to brain tissues via the bloodstream faces pure transport of substances across the blood–brain barrier (BBB). There are two types of drug delivery to brain tissues: systemic delivery through the bloodstream and local delivery via precise injections in the treatment area or using applications of specific drug carriers during surgical removal of the tumor. Different forms of materials are used in each way. Therefore, in the first case, only nanoscale particles or capsules with loaded drugs can be used [2]. In the second case, a polymer hydrogel implant could be placed in the resection cavity or the postoperative surface could be covered with a polymer film. In this way, a preliminary loading of the drug into the implanted material is carried out followed by its controlled release. Unfortunately, there are also difficulties with implantation into the affected area of the brain, which, in some cases, leads to the death of the patient [3].

In this regard, the development of drug delivery systems to the brain is one of the most urgent modern scientific topics. Biomaterials are versatile tools that can manage cell and tissue environments in vitro and in vivo, promoting the creation of well-defined macro and micro-environments. By combining bioengineering and chemical synthesis techniques, biomaterials can be designed and synthesized to specifically affect signal transduction pathways at various scales. They can also serve as carriers for therapeutic agents or modulate the microenvironment in vivo, leading to a direct therapeutic response against cancer cells and tumors [4].

Carbohydrate polymers are the main components of the extracellular matrix (ECM) of the human brain [5,6,7]. Therefore, biomaterials based on polysaccharides from different sources may be more convenient for the development of new therapeutic agents. The main advantages of carbohydrate polymers are low toxicity, biodegradability, biocompatibility and minimal immunogenicity. These features, combined with the ease of chemical modification and the low cost of raw materials, allow for the large-scale production of finely tuned therapeutic systems [8,9,10]. Carbohydrates are used to create various forms of materials, such as particles, films and hydrogels [11,12]. Carbohydrate biomaterials can also be specifically designed to deliver drugs and biologically active substances directly to the tumor cells. This can help to reduce side effects from medications and increase the effectiveness of treatment [13]. It was noted above that surgical removal of the brain tumor and implantation in this area is not effective in most cases. Nevertheless, research and development in this area is actively underway. Carbohydrate-based hydrogels can potentially be used to create implants that can be safely implanted into the patient’s body and remain there for a long period of time [14,15]. In addition, carbohydrate films can be specifically designed to control cancer growth and invasion [16] and to release biologically active substances that can inhibit tumor expansion [17,18]. Carbohydrate nanoparticles are potentially suitable to overcome the BBB because they stimulate transcytosis [19].

Pectin is a natural polymer and is one of the main components of the plant cell wall. The core pectin structure is composed of α-1,4-galacturonic acid residues partially esterified with methoxyl groups. These groups could be chemically engineered via alkaline de-esterification to produce a series of biopolymers characterized by different physico-chemical properties. Pectin-based materials are proposed for targeted drug delivery, wound healing and tissue engineering in biomedicine [20,21,22].

Pectins could be easily applied for the production of films, microparticles, microcapsules and fibers [23,24,25]. It is worth noting that there are many difficulties in the synthesis of pectin nanoparticles (NPs), as evidenced by the limited number of works. At the same time, pectin-based biomaterials could mimic the animal polysaccharides attributed to the components of ECM. Pectin helps to create a supportive microenvironment for neural cells, modulate cell migration and affect tissue-repair processes [8]. Moreover, chemical modifications of naturally produced pectin or improvements in the functional supplements give great opportunities for the development of pectin-based biomaterials targeted to diversified biomedical applications [8,25,26].

In this paper, we demonstrate the adjustment of the pectin degree of esterification (DE) provides for the development of biocompatible films and NPs suitable for prospective anticancer treatment, including nanocarriers that can penetrate the BBB, which is of interest for the therapy of gliomas, the most dangerous brain tumors. Special attention in the work is focused on the study of morphology, interfacial surface properties and the nanomechanical properties of films and NPs—all these parameters, as it was shown on other materials [27,28], should have a direct influence on tumor cell–ECM interactions.

## 2. Materials and Methods

### 2.1. Preparation of Modified Pectins

Preparations of modified pectins with DEs close to 0% and 50% were obtained from highly esterified citrus pectin with a DE of 60.2% (Copenhagen Pectin A/S, Lille Skensved, Denmark) by alkaline de-esterification, as was previously described [29]. Highly esterified citrus pectin was washed with ethanol and dried at 70 °C. After drying, the pectin sample was ground and fractionated according to the particle size. In the study, the fine fraction was sieved through a sieve with a cell size of 74 μm. The process of alkaline de-esterification of pectin performed at pH > 8.5 was preceded by the initial neutralization of free carboxyl groups of anhydrogalacturonic acid. When the desired DE was achieved, the reaction mixture was acidified with 1 M HCl solution in 50% ethanol, reaching a pH of 5–6 with vigorous stirring. The obtained pectin preparation was separated from the water–ethanol solution via filtration, first washed with 300 mL of a 50% ethanol solution and then with 150 mL of 95% ethanol. Washed pectin was dried at 70 °C. This method allowed us to obtain a set of products that differed in the content of methoxylated groups. Pectin sample characterization was performed according to the previously described technique [30].

### 2.2. Preparation of Modified Pectins Films

Powders of modified pectins with a DE of 0% (P0) and 50% (P50) were resuspended in deionized water and incubated for 1 h at room temperature for complete hydration. Concentrations from 0.5% to 1.5% by weight were used. Next, P0 samples were dissolved in a water bath with constant stirring for 1 h at 95 °C, P50 for 15 min at 60 °C. The resulting solutions were centrifuged at 3000× *g* for 40 min. The clarified colloidal solutions were transferred to new polypropylene tubes and autoclaved at 105 °C, 120 kPa for 10 min.

Plastic with an adhesive coating for cell culture was covered with the obtained solution of polysaccharides and left for 1 h for the adhesion of pectin molecules to the plastic. The unbound solution was removed and left to dry for 8 h at room temperature.

Gelation was initiated by binding pectin carboxyl groups with Ca^2+^ ions. Sterile gel initiator solutions were prepared based on 100 mM N-(2-hydroxyethyl)-piperazine-N’-2-ethanesulfonic acid (HEPES) pH 7.4, 300 mM NaCl and CaCl_2_ in an amount sufficient to complete gelation (Table 1). The dried films were incubated in a gel initiator solution for 40 min. After that, the excess initiator was removed and replaced with a complete nutrient medium.

### 2.3. Preparation of Modified Pectin Nanoparticles

Pectin solutions for NPs were prepared similarly to solutions for films but were not autoclaved. Solution of CaCl_2_ (gelling agent) was prepared by diluting 2 M solution to the concentration of 10 mM. NPs were produced by the interaction of polycations (Ca^2+^) with negatively charged carboxyl groups (ion gelation). For this aim, 200 μL of pectin was added to 1.5 mL of 10 mM CaCl_2_ solution in a drop-by-drop manner. Mixture was added to 2 mL test tube and treated in an ultrasonic bath (Elmasonic, Germany) under constant exposure at frequency 37 kHz. NPs were filtered through a sterile hydrophilic membrane filter with a pore size of 0.20 μm (Hyundai Micro, Korea) for in vitro testing.

### 2.4. Characterization of Modified Pectin Films

The surface morphology of hydrogel films and their viscoelastic properties were evaluated using a Bioscope resolve atomic force microscope (AFM; Bruker, Germany). Samples were scanned in PeakForce Tapping semi-contact mode with PFQNM-LC-A-CAL cantilever. The probe deflection sensitivity was calibrated with thermal noise method. Tip radius was set according to the manufacturer’s specification. Sample Poisson’s ratio was set at the value 0.5. All samples were analyzed in triplicate using Bruker Nanoscope analysis software (Version 1.40) to calculate the roughness, adhesion and elasticity values. The parameters such as arithmetic average height, which is the arithmetic average height parameter of the absolute values of the surface height deviations measured from the mean plane, and the root mean square average, which is the root mean square average of height deviations taken from the mean image data plane, were calculated. Before analysis, the films were incubated in complete Dulbecco’s Modified Eagle (DMEM) medium (Capricorn Scientific) for 12 h at 37 °C.

The measurement of the pectin film thickness was utilized by the recording of AFM Z step monitor value during the engagement to different regions of pectin film; after that, the gel layers were mechanically removed to measure the values for uncovered Petri dishes in the same regions. The differences between covered and uncovered Petri dishes were considered as gel thickness.

### 2.5. Characterization of Modified Pectin Nanoparticles

AFM measuring was carried out under the same conditions as for films. Prepared NPs were dripped onto 35 mm Petri dishes coated with poly-L-lysine into a place previously marked with a hydrophobic marker. The excess drops were removed after 30 min and ~2 mL of DMEM was added to the Petri dish. Morphology and mechanical property investigations were performed with parameters similar to those for films.

Zeta potential (ζ) was measured with a Zetasizer Nano ZS (Malvern Co., UK) using the Dynamic Light Scattering technique (DLS). The measurement was carried out in disposable polystyrene cuvettes at 25 °C.

FTIR studies were performed on an IRAffinity-1S (Shimadzu, Japan) with a PIKE technologies MIRacle 10 spectroscopy attachment for disturbed total reflection. All spectra were taken in absorption mode with Happ-Genzel apodization in the range of 400–4000 cm^−1^ with a resolution of 4 cm^−1^.

### 2.6. In Vitro Cell Proliferation Effect

Human malignant glioblastoma U87-MG cells were obtained from ATCC (Manassas, VA, USA) and cultured in DMEM/Ham’s F-12 supplemented with L-glutamine (Capricorn), 10% fetal bovine serum (Gibco), 100 U/mL penicillin and 100 μg/mL streptomycin at 37 °C and CO_2_ content 5%. The medium was changed every 72 h. Cells were harvested after reaching 70% confluency, followed by washing with 1× PBS buffer, resuspending, and using for experiments.

Model adhesive cell line was cultured on pectin-based substrates with DEs of 0% and 50% at concentrations of 0.5%, 1% and 1.5% to study the effect of pectin hydrogel substrates on the behavior of U87MG glioblastoma cells. In addition, suspensions of NPs with different contents of pectin, 0.5%, 1% and 1.5%, were added to U87MG cells cultured on plastic to study the effect of pectin NPs.

Integrated platform for continuous monitoring of live cells in culture Cell-IQ (CM Technologies Oy, Finland) was used to obtain data on cell proliferative activity. Cells were seeded on hydrogel substrates or plastic at a density of 7.5 × 10^3^ cells/cm^2^ and cultured in complete DMEM/F12 nutrient medium for 5 days at 37 °C, 5% CO_2_. Cells in culture were visualized using the phase contrast method. Imaging of each field of view was carried out with an interval of 4 h. For each material, cells were observed in 25 fields of view using a Nikon Plan Fluor 10× objective with a numerical aperture of 0.30. The number of cells in each field of view was determined automatically using the Cell-iQ Analyzer program.

Population doubling time (DT) was determined using the Malthusian growth model by:

P(t) = P_0_e^rt^, (1)

where P_0_ = P(0) is the initial population size, r = the population growth rate (Malthusian parameter of population growth) and t = time.

### 2.7. Statistical Analysis

Statistical analyses were performed via GraphPad Prism software (GraphPad Software, USA). Statistical differences were designated significant if *p*-values were less than 0.05 (* *p* ≤ 0.05) and highly significant if *p*-values were less than 0.01 (** *p* ≤ 0.01), less than 0.001 (*** *p* ≤ 0.001) or less than 0.0001 (**** *p* ≤ 0.0001). The Mann–Whitney U-test was used to quantify differences between the two groups. One-way analysis of variance (ANOVA) was used to compare three or more groups. Fractions with a percentage ratio of more than 5% of the total distribution were used to calculate the average particle size and standard deviation.

The raw data were fitted using non-linear regression exponential (Malthusian) growth and the best-fit values were obtained using least squares regression fitting method to quantify the differences between the cell culture proliferation rates and find the cell doubling times.

## 3. Results

### 3.1. Characterization of Modified Pectin Films

#### 3.1.1. Films Morphology

**Figure 1:**
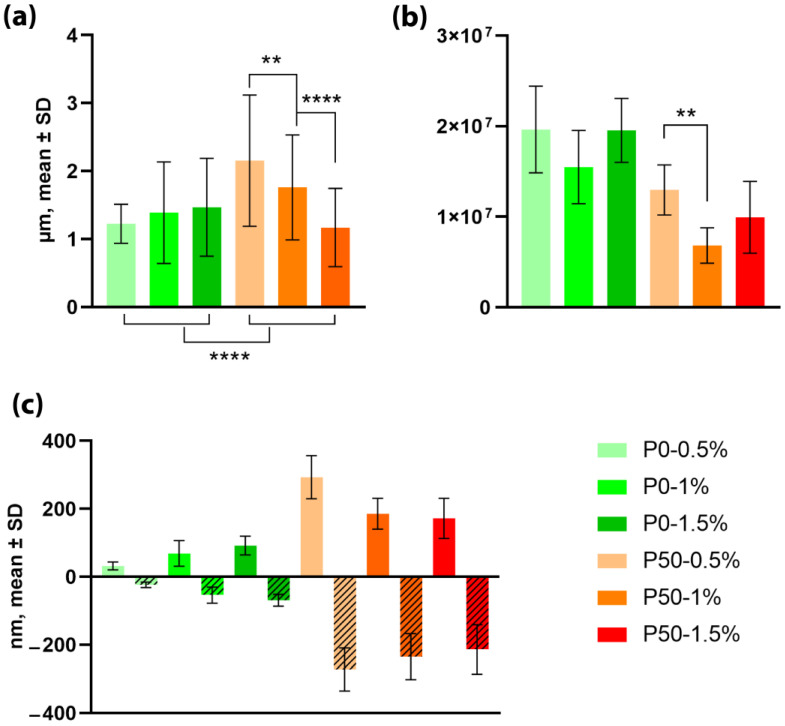
Morphology of modified pectin-based films: (**a**) pore size, (**b**) number of pores per 1 cm^2^, (**c**) roughness of films, (*n* = 100); ** *p* value < 0.01; **** *p* value < 0.0001.

The pore size of films (Figure 1a) based on P0 hydrogels at different concentrations of pectin did not differ significantly: 1.57 ± 0.6 µm, 1.41 ± 0.74 µm and 1.4 ± 0.67 µm for P0-0.5%, P0-1% and P0-1.5%, respectively. At the same time, the differences in the number of pores per field of view for these films (Figure 1b) were not statistically significant, although each field of view of P0-1% accounted for an average of 35 pores, which is 21.5% less compared with P0-0.5% and P0-1.5% (44 pores per field of view).

Statistically significant dependence of the pore size on the concentration of pectin was observed for films based on P50. With an increase in the concentration from 0.5% to 1.5%, the pore size decreased from 2.15 ± 0.96 μm to 1.14 ± 0.57 μm, while the number of pores per field of view was noticeably smaller for the P50-1% film: 15 compared with 29 for P50-0.5% and 22 for P50-1.5%.

At the same time, the height differences for P0 were noticeably smaller than for P50 (Figure 1c). The difference between maximum and minimum heights (roughness) of the analyzed surface increased from 55.32 nm to 161.04 nm with the increasing concentration of P0 films. An inverse relationship was observed for P50 films: the height difference decreased from 565.3 nm to 385.95 nm with the increasing concentration. The deepest pores were observed in P50-0.5% samples, 272.44 ± 63.37 nm, and the most superficial ones in P0-0.5% samples, 23.4 ± 7.97 nm.

#### 3.1.2. Viscoelastic Properties of Films

**Figure 2:**
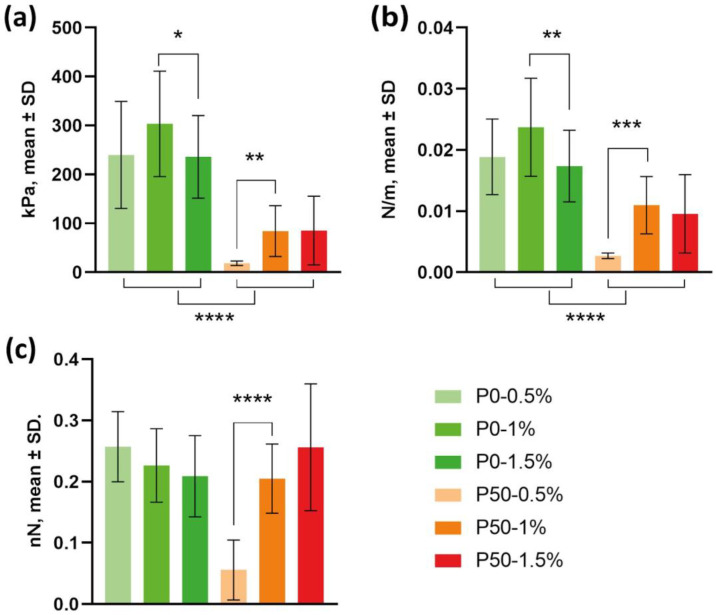
Mechanical properties of modified pectin-based films: (**a**) elasticity (Young’s modulus), (**b**) stiffness, (**c**) adhesiveness, (*n* = 6); * *p* value < 0.05; ** *p* value < 0.01; *** *p* value < 0.001, **** *p* value < 0.0001.

AFM was used to determine the Young’s modulus (E), stiffness and adhesiveness of thin nanofilm surfaces. This made it possible to characterize the mechanical properties of films based on modified pectins at the molecular level. The values of the Young’s modulus of the films (Figure 2a) made of P0 were significantly higher than those for P50 and did not depend on the concentration of pectin: for P0-0.5%—E was 239.8 ± 109.4 kPa, for P0-1%—303.4 ± 107.8 kPa, and for P0-1.5%—235.9 ± 84.4 kPa. Differences in E values for these samples were not statistically significant. Differences in the Young’s modulus values for films from P50-1% and P50-1.5%, which amounted to 84.2 ± 51.9 kPa and 85.3 ± 70.2 kPa, respectively, were also not reliable. The average value of E for low-concentration P50 films was much lower: 18.6 ± 4.7 kPa.

The rigidity of the modified pectin films (Figure 2b) turned out to be similar to the elastic modulus: the P0 materials turned out to be the hardest, and their stiffness varied from 0.0189 ± 0.0062 N/m for P0-0.5% and 0.0174 ± 0.0059 N/m for P0-1.5% to 0.0237 ± 0.008 N/m for P0-1%. The differences were not statistically significant. The hardness of the film from P50-0.5% turned out to be significantly lower than that of P50-1% and P50-1.5%: 0.0027 ± 0.0005 N/m vs. 0.011 ± 0.0047 N/m and 0.0097 ± 0.0065 N/m, respectively.

The adhesiveness of the probe to the analyzed films (Figure 2c) did not change significantly and ranged from 0.2049 ± 0.0565 nN to 0.2572 ± 0.0573 nN, except for samples P50-0.5%. Adhesion to these films was 0.0558 ± 0.0489 nN.

**Figure 3:**
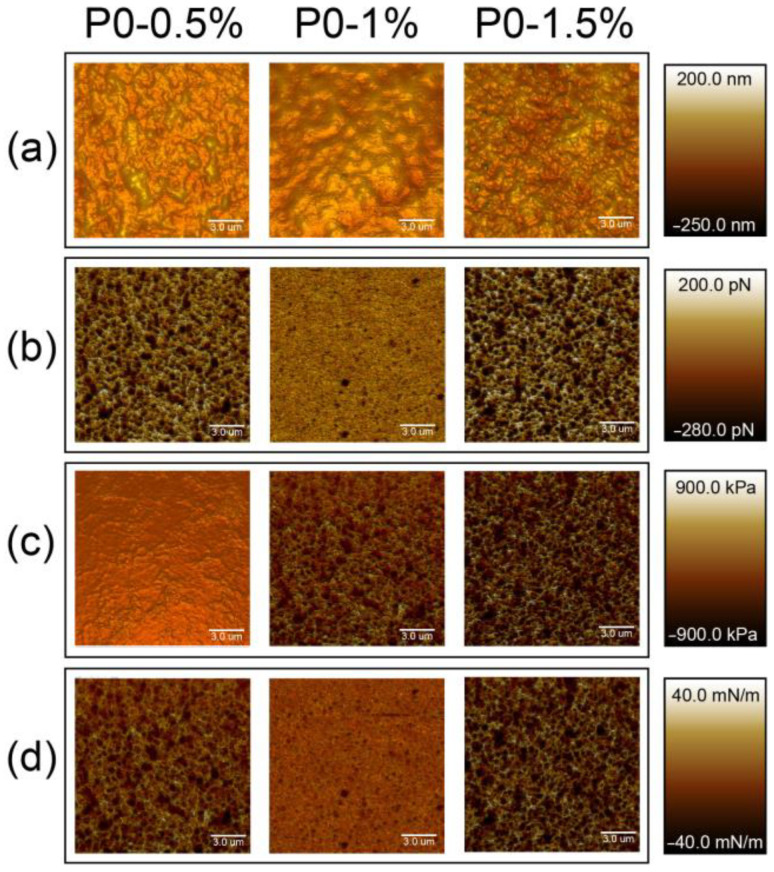
AFM images of modified pectin-based films (DE 0%): (**a**) topographic morphology, (**b**) adhesiveness, (**c**) Young’s modulus, (**d**) stiffness.

**Figure 4:**
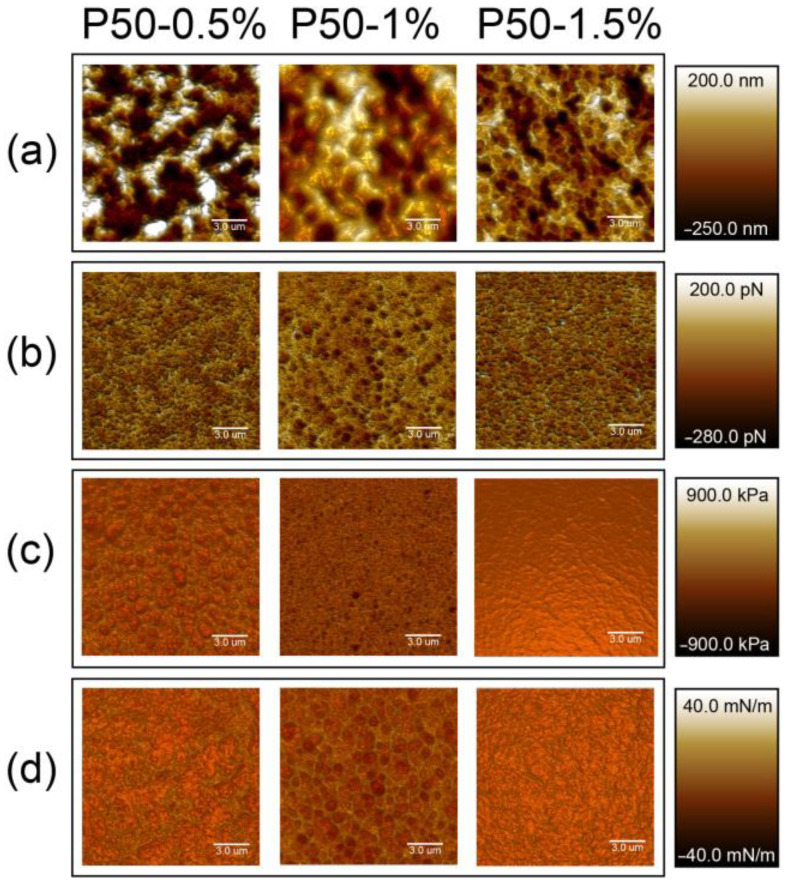
AFM images of modified pectin-based films (DE 50%): (**a**) topographic morphology, (**b**) adhesiveness, (**c**) Young’s modulus, (**d**) stiffness.

Figure 3 and Figure 4 show the superimposition of the morphology (a), adhesiveness (b), modulus of elasticity (c) and stiffness (d) on the topographic morphology of the pectin-based films. Films have heterogeneous porous structures, the typical morphology of which is shown in Figure 3a and Figure 4a. The scale bar reflects the height of the sample surface. In other cases (b–d), the scale reflects the degree of adhesion of the AFM probe to the sample surface and the ability of the material to resist elastic deformation, respectively. Thus, the brightest areas correspond to the highest values of the analyzed parameters.

### 3.2. Characterization of Modified Pectin Nanoparticles

#### 3.2.1. Nanoparticles’ Morphology

**Figure 5:**
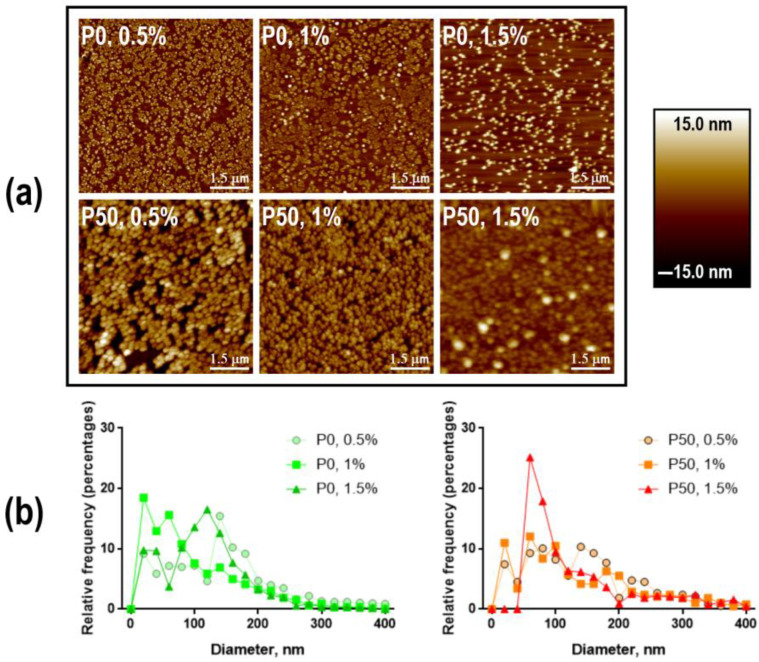
Surface morphology (**a**) and size distributions (**b**) of modified pectin NPs from AFM. P0—pectin with DE 0%, P50—pectin with DE 50%.

According to the AFM results, the NPs had a round shape (Figure 5a). Figure 5b shows the size distribution of the NPs. The mean diameters of NPs based on pectin with a DE of 0% were 99.03 ± 44.28 nm for P0-0.5%, 88.59 ± 41.44 nm for P0-1% and 116.80 ± 38.31 nm for P0-1.5%. NPs based on pectin with a DE of 50% had an average size of 114.40 ± 44.23 nm, 115.50 ± 51.92 nm and 92.76 ± 34.46 nm for P50-0.5%, P50-1% and P50-1.5%, respectively. In both cases (P0 and P50), there was no exact dependence of the particle size on the concentration and DE of the polysaccharide.

#### 3.2.2. Mechanical Properties of Nanoparticles

**Figure 6:**
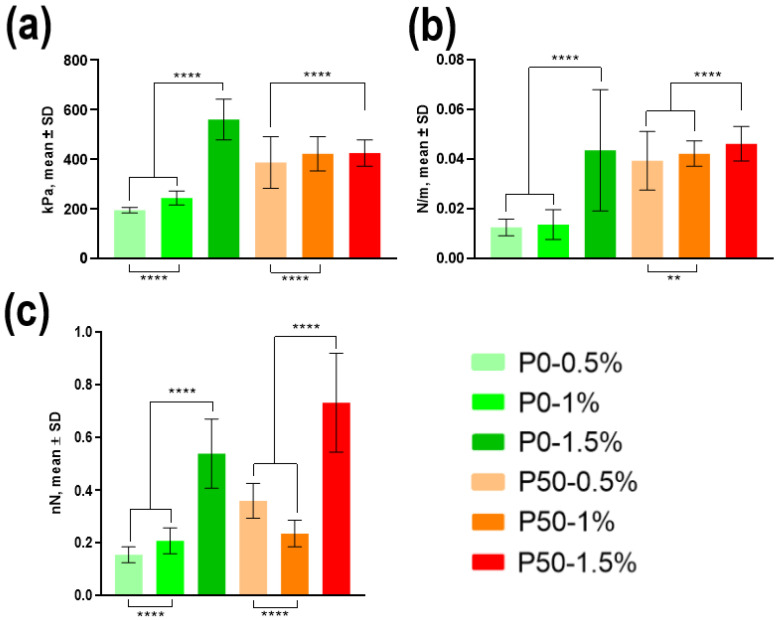
Mechanical properties of modified pectin-based NPs: (**a**) elasticity (Young’s modulus), (**b**) stiffness, (**c**) adhesiveness. (*n* = 6) ** *p* value < 0.01; **** *p* value < 0.0001.

The AFM was used to determine the Young’s modulus (E), stiffness and adhesiveness of NPs (Figure 6). The Young’s modulus for P0 NPs (Figure 6a) was 196.1 ± 11.4 kPa for 0.5%, 245.1 ± 28.3 kPa for 1% and 561.2 ± 81.7 kPa for 1.5%. P50 samples demonstrated the following values: 388.2 ± 104.0 kPa for 0.5%, 423.6 ± 69.0 kPa for 1% and 426.3 ± 52.7 kPa for 1.5%. Young’s modulus increased with the change in pectin concentration from 0.5% to 1.5% for P0. No significant changes in the elastic modulus for different concentrations of pectin P50 were found.

The stiffness of P0 NPs (Figure 6b) was equal to 0.0127 ± 0.0033 N/m, 0.0138 ± 0.006 N/m and 0.0437 ± 0.0244 N/m for concentrations of 0.5%, 1% and 1.5%, respectively. The values of this parameter were 0.0395 ± 0.0118 N/m, 0.0424 ± 0.0051 N/m and 0.0464 ± 0.007 N/m for similar concentrations in the case of P50. Stiffness also increased along with the concentration of pectin, with some deviations. There was no difference for samples P0 0.5% and 1%.

**Figure 7:**
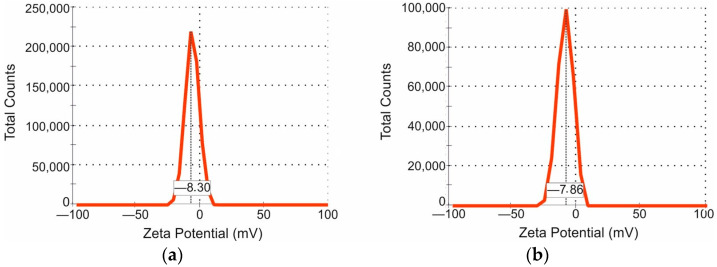
Zeta potential distribution of modified pectin NPs from Zetasizer Nano ZS: (**a**) P0, 0.5%; (**b**) P50, 0.5%.

Adhesiveness studies of P0 samples have shown values (Figure 6c) of 0.16 ± 0.03 nN for 0.5%, 0.21 ± 0.05 nN for 1% and 0.54 ± 0.13 nN for 1.5%. The adhesiveness of P50 samples was 0,36 ± 0.07 nN, 0.24 ± 0.05 nN and 0.73 ± 0.19 nN for similar concentrations. We can note the general dependence of the increase in adhesiveness with the growth in the concentration of P50 samples. The results of zeta potential measurements are demonstrated for NP samples with pectin concentrations of 0.5% by mass (Figure 7). They show approximately identical results for P0 and P50: −8.30 and −7.86 mV, respectively. 

#### 3.2.3. FTIR Spectroscopy of Nanoparticles

**Figure 8:**
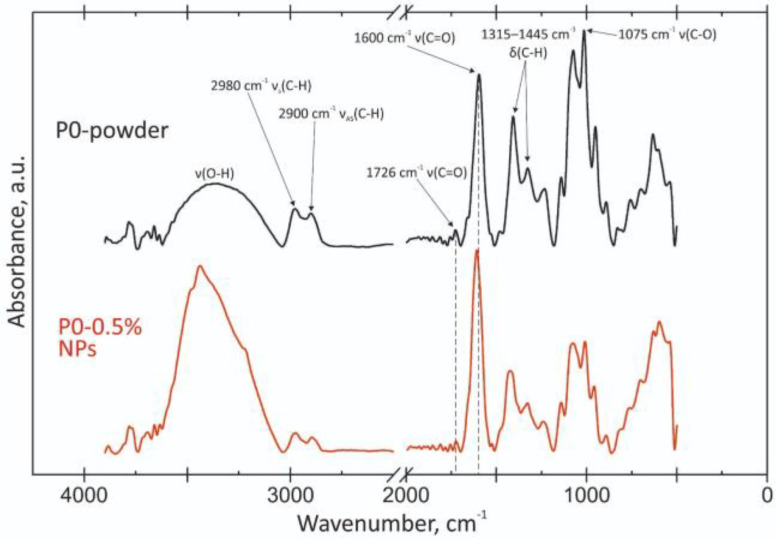
FTIR of modified pectin-based NPs from pectin with DE 0%.

**Figure 9:**
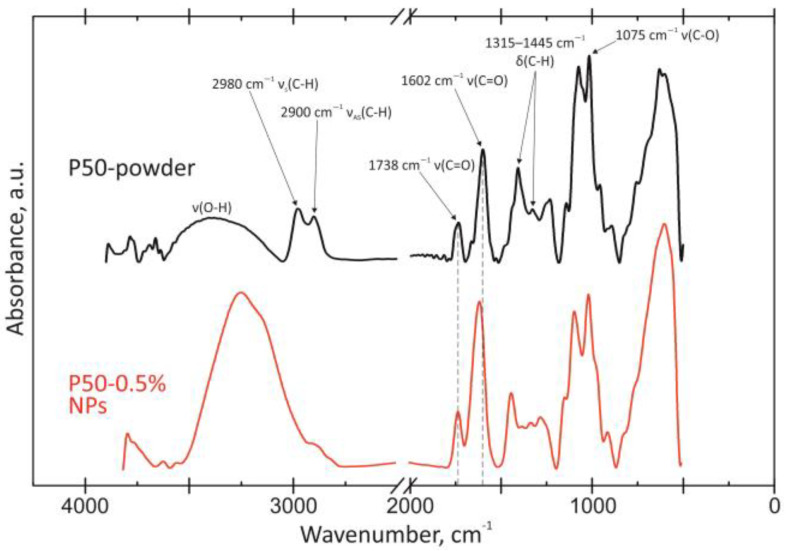
FTIR of modified pectin-based NPs from pectin with DE 50%.

Figure 8 and Figure 9 show FTIR spectra of pectin with a DE of 0 (P0) and 50 (P50), as well as NPs obtained from these biopolymers. The spectrum of the original polysaccharide P0 contains characteristic absorption bands at 3385, 2980/2900, 1408/1325 and 1075/1012 cm^−1^. They are attributed to the vibrations of ν(O-H), ν(C-H), δ(C-H) and ν(C-O), respectively. For pectin P50, these bands are located at 3376, 2980/2900, 1412/1317 and 1075/1015 cm^−1^. The spectra of the initial polysaccharides were correlated with the article [31].

The bands on both spectra at 1726 (very weak) and 1738 cm^−1^ correspond to ν(C=O) esterified carboxyl vibrations of both pectins, 1600 and 1602 to ν_as_(C=O)—ionized carboxyl (-COO_2_) polysaccharides in salt form. The bands at 1664 and 1660 cm^−1^ in some articles refer to variations in the acidic residues in commercial pectin samples. The DE of pectins 0 and 50 were calculated based on the method from the article [32]. Approximate results of ~0% and ~35% were obtained. Despite the large difference in the case of P50, the chemical confirmation methods showed results close to those stated.

The characteristic bands of pectins during NP formation remain almost unchanged. It is worth noting that the position of ν(C=O) esterified carboxyl does not change. The ν_as_(C=O) ionized carboxyl (-COO_2_) vibration band does not change in intensity and shifts to the low-frequency region by less than 15 cm^−1^. Such changes can be classified as insignificant. The region of ν(O-H) vibrations at high frequencies significantly changes its shape and increases in intensity. It includes vibrations not only of water, but also of hydrogen bonds, which may also be the cause of NP formation.

### 3.3. Biocompatibility of Modified Pectin Films and Nanoparticles

#### 3.3.1. Effect on Metabolic Activity of Cells

**Figure 10:**
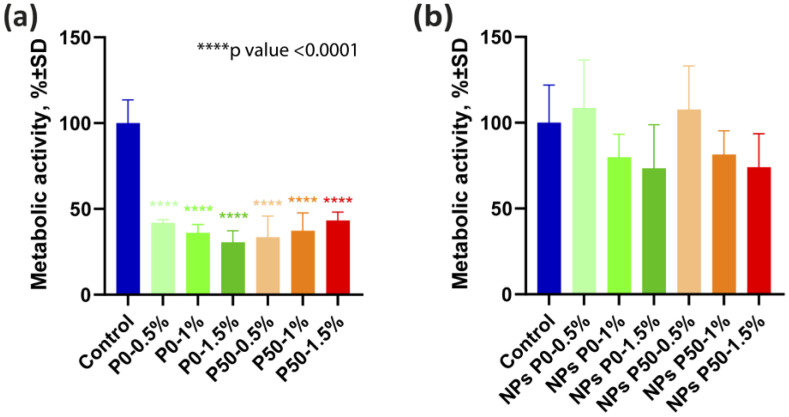
Metabolic activity of U87MG cells after 72 h cultivation. (**a**) on pectin-based films, (**b**) on plastic with additional pectin-based NPs. *n* = 8, *p* < 0.0001.

We evaluated the ability of pectin-based films and NPs to reduce the metabolic activity of U87MG human glioblastoma cells using the MTT assay (Figure 10). Pectin-based films (Figure 10a), regardless of the content of polysaccharide and its DE, significantly (*p* < 0.0001) inhibited the metabolism of glioblastoma cells relative to cells cultured on plastic up to 42.0%, 36.1% and 30.6% (P0- 0.5%, P0-1% and P0-1.5%, respectively) and up to 33.5%, 37.3% and 43.3% (P50-0.5%, P50-1% and P50-1.5%, respectively). All studied NPs did not significantly change the metabolic activity of the cells (Figure 10b).

#### 3.3.2. Effect on Cell Proliferation

**Figure 11:**
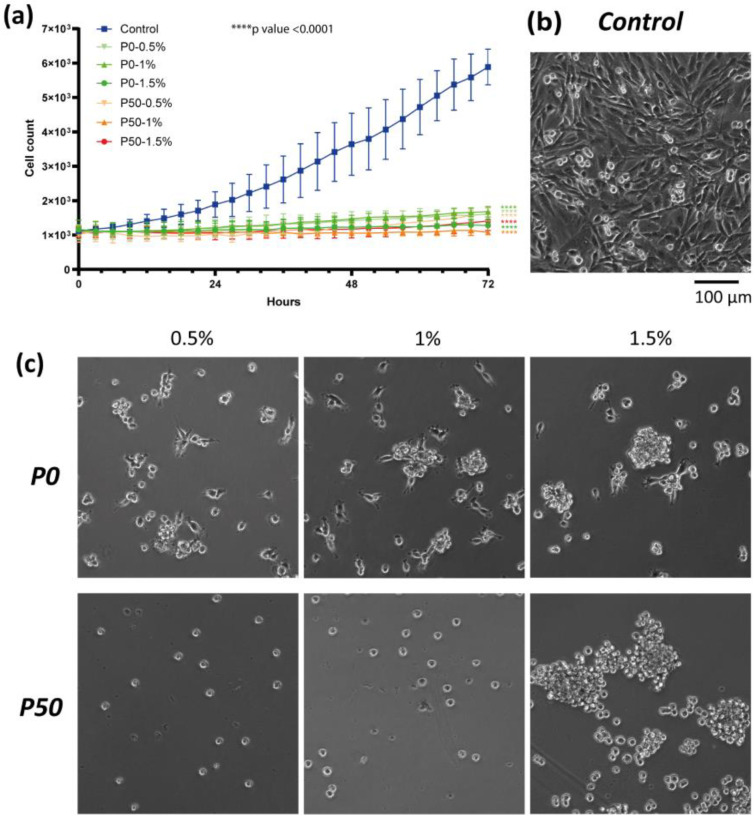
Effect of pectin-based films on U87MG glioblastoma cells during 72 h of cultivation: (**a**) growth curve, *p* < 0.0001, (**b**) morphology of U87MG cells on polystyrene after 72 h, (**c**) morphology of U87MG cells on pectin-based films after 72 h.

The analysis of the cells’ number and their behavior was carried out for 3 days. It has been established that the morphology of U87MG glioblastoma cells during their cultivation on polystyrene and hydrogel substrates differs significantly. The cells spread out and have a triangular and fusiform shape with neurites on a standard polystyrene coating for adhesive cultivation, while cells grow clonally and form rounded neurosphere-like agglomerates on both types of film substrates (Figure 11). This indicates a reduced ability of cells to migrate and adhere to the substrate. At the same time, cells are characterized by a spherical morphology on pectin substrate with a DE of 50%, and U87MG glioblastoma cells have outgrowths on substrates of de-esterified pectin.

**Figure 12:**
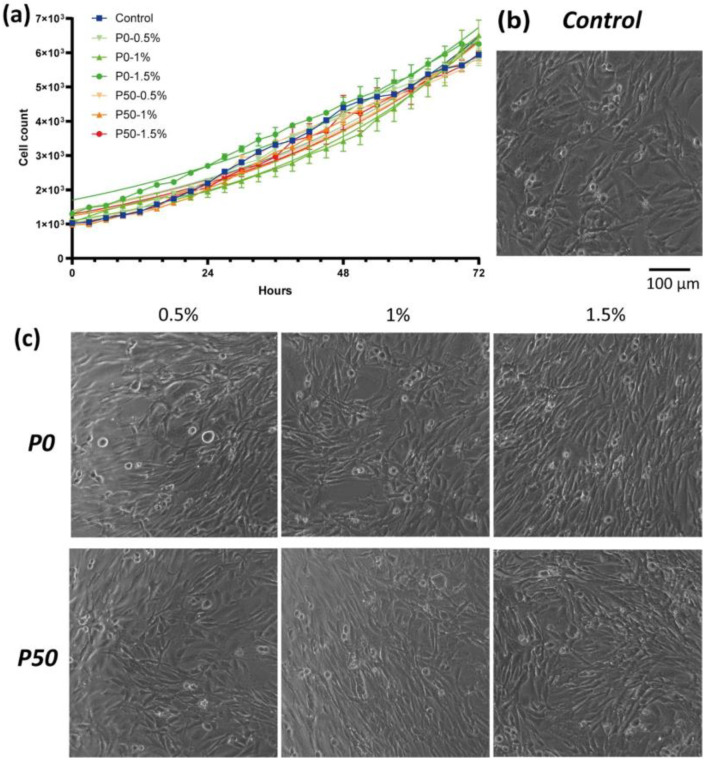
Effect of pectin-based NPs on U87MG glioblastoma cells during 72 h of cultivation: (**a**) growth curve, (**b**) morphology of U87MG cells on control after 72 h, (**c**) morphology of U87MG cells 72 h after addition of pectin-based NPs.

The number of cells cultivated on pectin substrates almost did not change over time. The number of U87MG cells increased 1.6-, 1.5- and 1.1-fold on the films P0-0.5%, P0-1% and P0-1.5% and 1.9-, 1.0- and 1.3-fold on the films P50-0.5%, P50-1% and P50-1.5% after 72 h of cultivation, respectively, while in the control, the number of cells increased 5.5-fold relative to the beginning of the experiment. The addition of pectin-based NPs did not affect U87MG cells. In all samples and controls, cells retained an unchanged triangular or fusiform morphology (Figure 12). The number of U87MG cells increased 5.6-, 6.2- and 4.8-fold for P0-0.5%, P0-1% and P0-1.5% and 5.7-, 6.0- and 5.8-fold for P50-0.5%, P50-1% and P50-1.5% after 72 h of cultivation, respectively.

The doubling time of the cell population in the control was 29.2 h, while the doubling time on pectin-based films with a DE of 0% was 114.6 h, 105.3 h and 310.3 h for P0-0.5%, P0-1% and P0-1.5%, respectively. The doubling time of the cell population on P50-0.5% and P50-1.5% samples was 95.5 h and 190.0 h, respectively. The number of cells on the P50-1% film did not change significantly during the experiment. The doubling time of the cell population in the experiment was close to that of the control group and amounted to 32.7, 28.2 and 36.2 h for P0-0.5%, P0-1% and P0-1.5% NPs and 32.7, 30.2 and 31.3 for P50-0.5%, P50-1% and P50-1.5%, respectively.

## 4. Discussion

Pectins are easily soluble in basic media and can participate in gelation processes due to the presence of ionogenic carboxyl groups. Some of them are naturally esterified by a conversion to methoxylated derivatives. Biomaterials obtained from pectin gels could be used in diversified biomedical applications. Coatings are of interest for the safety of medical devices because they inhibit the growth of pathogenic microorganisms on their surface [33]. In addition, pectin-based hydrogels and films with the addition of other polymers are good candidates for wound dressings and treatments due to their water-retention ability, toxin absorption properties, biodegradability and biocompatibility [34,35,36,37,38]. Another application of pectin biomaterials is in the development of polymer coatings with tuned surface properties that promote bone growth. It is very important to increase the lifetime of load-bearing implants [39].

Modification by functional group rearrangement makes it possible to expand the application areas of pectin-based biomaterials, including the design of biocompatible and harmless carriers of drugs [40]. A lack of toxicity, resistance to proteases and amylases and the ability to be degraded by intestinal microflora make it very interesting for obtaining controlled-release dosage forms for orally administered drugs. For these purposes, pectin hydrogel structures are used as binders in gel bead and granule formulations [40,41]. Pectin-based biomaterials have been investigated for the treatment of colorectal diseases and gastrointestinal cancers. Pectin coatings can provide protection of the drug in the upper gastrointestinal tract and enzymatic cleavage and release of the drug in the colon. For example, oral administration of solid bilayer lipid particles coated with pectin and loaded with soluble curcumin showed high efficiency in delivering a soluble drug to the tumor localization site [42]. In another case, a graphene-chitosan oxide nanocomposite coated with pectin for the delivery of drugs directed to the colon has selectively eliminated cancer cells, which indicates that it is a promising therapeutic agent for cancer treatment [43]. Also, pectin biomaterials conjugated with specific agents such as DOX [44,45] and MTX [46] showed antitumor efficiency of the polysaccharide against different types of tumors.

There are just a few reports about the application of pectin biomaterials in glioblastoma or glioma treatment. For example, the addition of pectin fraction to cells reduced the number of adherent glioblastoma cells. The number of adherent U251-MG cells decreased to 37.65% and the number of adherent T98 G cells decreased to 35.55%. The cytotoxic effect was also confirmed with an MTT assay with similar results [47]. Low-esterified citrus pectins with DEs from 27 to 40% were used to produce pectin-coated etoposide and olaparib NPs as part of a bioadhesive hydrogel. The in vitro biocompatibility assay of pectin with a DE of 35% measured by relative metabolic activity showed the absence of toxicity in the U87 GBM cell line for 72 h upon exposure to 50–200 μM pectin gels [48].

The possibility of modulating the behavior of glioblastoma cells using hydrogels based on modified pectins and extracellular matrix proteins has already been shown in our previous article [8]. In this work, we have elaborated pectin films and NPs for prospective anticancer therapy. Our pectin-based films showed a 5-fold decrease in the number of U87MG cells compared with the control after 72 h of cultivation. Moreover, the number of cells cultivated on pectin substrates almost did not change over time. The MTT-assay results demonstrated the inhibition of the metabolism of glioblastoma cells relative to cells cultured on plastic up to 31%. Additionally, the absence of neurites of cultivated U87MG glioblastoma cells indicate a reduced ability of cells to migrate and adhere to the substrate. Due to these facts, we can suppose pectin-based films have an inhibitory effect on glioblastoma cells even without loading of antitumor agents. It is interesting to note that the addition of pectin-based NPs did not affect U87MG cells. This difference in the obtained results for pectin biomaterials requires further research, which will be carried out in subsequent works. However, the lack of toxicity in the case of NPs, as well as further work to increase specific targeting towards tumor cells, will reduce the harmful effects on healthy cells.

The production of nanosized particles is determined by the way of their delivery to the brain. The obtained pectin NPs ranged in size from 30 to 400 nm, with more than half of the particles up to 200 nm (Figure 5b). Such dimensions are observed for many polymeric NPs produced by various methods for the pharmaceutical industry, even already loaded with bioactive agents [48,49,50]. It has not previously been achieved for pectin particles. This advantage makes them suitable for use as a nanostructured delivery system to the brain for anticancer purposes. The particle size capable of passing through the BBB may be as small as 200 nm. Most of the particles will be taken up through caveolin-mediated endocytosis and the remaining part through clathrin-mediated endocytosis [51]. Since the enzymes for effective pectin degradation are only present in the gastrointestinal microbiota, there is no danger of NPs entering intracellular lysosomes. However, further experimental research needs to clarify pectin degradation under enzymes originating from mammalian organisms. In addition, the surface charge of the obtained particles has a negative value (from −8.30 to −7.86 mV). This property could be utilized for the immobilization of other molecules that are of interest for the production of pectin nanoparticles supplemented with drugs and specific protein ligands targeted to the molecules exposed on the cancer cell surface.

Nanomechanical properties of pectin films and NPs are crucial for the effect on tumor cells, which was demonstrated by the example of hydrogels in our recent work [8]. The films obtained were found to be quite soft. The Young’s modulus for P0 ranged from 239.8 ± 109.4 kPa to 303.4 ± 107.8 kPa. P50 was even softer, ranging from 18.6 ± 4.7 kPa to 85.3 ± 70.2 kPa. It is noteworthy that even such soft films showed stable mechanical properties and the ability to maintain and affect the cells for a long time. The mechanical property result we obtained could not be compared with those found in the literature, as the results are highly variable. This can be attributed to the difference in film production and investigation methods. Thus, Aleksandra Nesić et al. prepared much stiffer pectin-based films with a DE of 60% for antibacterial coating of biomedical equipment by crosslinking with zinc ions. The Young’s modulus of these films was 4930 ± 5% MPa [33]. Studies on the mechanical properties of pectin-based composite films using the stretch-to-break method were carried out in [52]. The materials were investigated in relation to the HT-29 colon cancer cell line. The difference from our work is that one type of pectin was used, which interacted with three different nucleobase (NB) units (cytosine, thymine, uracil). Nevertheless, it was found that the cytotoxicity and apoptotic activity in HT-29 increased with the increasing of tensile strength and elongation-at-break.

The nanomechanical properties of pectin NPs have also been investigated. Several examples were found in the literature. The absorption effect of PEG-PLA micelles of ~17 nm size with a Young’s modulus of 260 kPa (rigid) and 165 kPa (soft) by human melanoma cell line A375 was demonstrated in [53]. When the NP size was increased to ~75 nm, the absorbance also increased. In another work for the HeLa cell line, PEGylated polymer-lipid NPs with a Young’s modulus of 1.2 GPa absorbed better than the same NPs with a Young’s modulus of 0.76 GPa [54]. It cannot be said that mechanical properties contribute more to cellular uptake than chemical modification of the surface or size, but other things being equal, hard particles have a higher penetration efficiency than soft particles. This is due to the fact that hard NPs expend less energy to envelope their membrane during endocytosis [55]. For example, for the breast cancer cell line 4T1, no differences were found in the uptake of polymeric hydrogel NPs with a Young’s modulus of 10 kPa and 3000 kPa at the same size of ~200 nm. When their surface was modified with antibodies against ICAM, the absorption increased significantly, and it was statistically higher for hard than for soft [56].

The Young’s modulus of the NPs obtained in our work ranged from 196.1 ± 11.4 kPa to 561.2 ± 81.7 kPa. Approximately the same values were obtained by Anselmo et al. [56] with polymer hydrogel (PEG) NPs. The authors showed that NPs with a Young’s modulus of 10 kPa penetrated cells almost as well as particles with a Young’s modulus of 3000 kPa. The difference was only apparent after 12 h of incubation, and NPs with a Young’s modulus of 3000 kPa penetrated cells 1.2 times more efficiently. When the NPs were modified with an antibody, they began to penetrate about 1.5 times more efficiently. Moreover, the modified particles with a Young’s modulus of 3000 kPa penetrated better than those with a Young’s modulus of 10 kPa. Thus, it can be assumed that the pectin NPs obtained in our work will pass through the BBB and be taken up by cancer cells. Moreover, NPs with a Young’s modulus of 561.2 kPa (P0-1.5%) should pass through and be absorbed more actively than those with a Young’s modulus of 196.1 kPa (P0-0.5%).

## 5. Conclusions

The aim of this work was to develop prospective biomaterials for drug delivery systems in anticancer therapy. Hydrogel films and NPs based on plant-derived polysaccharides pectins were obtained by varying the set of free carboxyl groups (DE) and polymer concentration. The structure of pectin-based films, their physico-chemical properties, biocompatibility and the ability to inhibit the growth of the U87MG human glioblastoma cell line make these biomaterials promising for intraoperative processing when surgically removing brain tumors. The use of the ion gelation protocol in combination with a relatively simple biopolymer injection into cross-linker agent pectin-based NPs was elaborated and diversely characterized. NPs displayed a lack of general toxicity when tested on a human glioma cell culture, which is, rather, a benefit and suitable for loading with a specific drug targeted to cancer cells only. NPs’ shape, average dimensions, surface charge and nanomechanical properties were investigated and proved to be suitable non-invasive drug delivery systems, which can be improved with protein molecules recognizing specific types of tumor cells.

## Figures and Tables

**Table 1:** Ca^2+^ content in the gel initiator solution.

Sample	Ca^2+^ (mM)
P0-0.5%	1.33
P0-1%	2.66
P0-1.5%	4
P50-0.5%	4
P50-1%	8
P50-1.5%	12

## Data Availability

Images and data are available from the corresponding author upon reasonable request.

## Abbreviations

AFM	atomic force microscope
BBB	blood–brain barrier
DE	esterification degree
DMEM	Dulbecco’s Modified Eagle Medium
FTIR	Fourier-transform infrared spectroscopy
HA	hyaluronic acid
HEPES	N-(2-hydroxyethyl)-piperazine-N’-2-ethanesulfonic acid
NP	nanoparticle
P0	pectins with an esterification degree of 0%
P50	pectins with an esterification degree of 50%
